# Supplementary material for: Elementary effects for models with dimensional inputs of arbitrary type and range: Scaling and trajectory generation
Source: PLoS One. 2023 Oct 25;18(10):e0293344. doi: 10.1371/journal.pone.0293344 (PMC10599590; doi:10.1371/journal.pone.0293344)
Supplement: S1 File — (PDF) [file pone.0293344.s001.pdf]

Appendices

S1 Elementary effects

An elementary effect is typically defined as

$$ee^n_{ij} = \frac{Y_j(\mathbf{x} + \boldsymbol{e}_i\delta_i) - Y_j(\mathbf{x})}{\delta_i}, \tag{S1}$$

where $\delta_i \in \{1/(p_i - 1), 2/(p_i - 1), \dots, 1\}$. However, this does not properly account for steps in the negative direction, in which case the effect is actually

$$ee^n_{ij} = \frac{Y_j(\mathbf{x}) - Y_j(\mathbf{x} - \boldsymbol{e}_i\delta_i)}{\delta_i}. \tag{S2}$$

Both expressions can however be combined into a single equation

$$ee^n_{ij} = \frac{Y_j(\mathbf{x} + \boldsymbol{e}_i\delta_i) - Y_j(\mathbf{x})}{\delta_i}, \tag{S3}$$

by letting $\delta_i \in \{\pm 1/(p_i - 1), \pm 2/(p_i - 1), \dots, \pm 1\}$. If the step is in the positive direction (so $\delta_i \in \{1/(p_i - 1), 2/(p_i - 1), \dots, 1\}$), Equation (S3) equals Equation (S1). On the other hand, for a step in the negative direction (so $\delta_i \in \{-1/(p_i - 1), -2/(p_i - 1), \dots, -1\}$), Equation (S3) can be written as

$$ee^n_{ij} = \frac{Y_j(\mathbf{x}) - Y_j(\mathbf{x} - \boldsymbol{e}_i|\delta_i|)}{|\delta_i|} \tag{S4}$$

which equals Equation (S2).

S2 Discrepancy

Following Morokoff and Caflisch [1], discrepancy of a sequence $\{\mathbf{x}^{(i)}\}_{i=1}^N \subset [0,1]^d$ centers around the quantity

$$R_N(J) = \frac{1}{N}\#[\mathbf{x}^{(i)} \in J] - \mathrm{Vol}(J), \tag{S5}$$

where $J \subseteq [0,1]^d$, $\#[\mathbf{x}^{(i)} \in J]$ is the number of points in J and $\mathrm{Vol}(J)$ is the volume of J. $R_N(J)$ gives the deviation of the sequence from complete uniformity in the sub-region J. Different kinds of discrepancies can then be obtained by restricting the sub-region J to a certain class of sets and by taking a certain norm of R_N over this class [1].

The L_∞ (or sup) and L_2 discrepancy of a sequence $\{\mathbf{x}^{(i)}\}_{i=1}^N$ are defined as

$$D_\infty(N,d) = \sup_{\mathcal{I} \subset E} \left| \frac{1}{N} \#[\mathbf{x}^{(i)} \in \mathcal{I}] - \mathrm{Vol}(\mathcal{I}) \right| \tag{S6}$$

and

$$T_2^2(N,d) = \int\limits_{\substack{[0,1]^{2d}, \\ y_i < z_i}} \left[\frac{1}{N} \#[\mathbf{x}^{(i)} \in [\mathbf{y},\mathbf{z}] - \mathrm{Vol}([\mathbf{y},\mathbf{z}]) \right]^2 d\mathbf{y} d\mathbf{z}, \tag{S7}$$

respectively, where $\mathrm{Vol}(V)$ is the volume of region V and E is the set of all sub-rectangles of $[0,1]^d$. Similarly, the star variants ($D_\infty^\star$ and $T_2^\star$) are defined by restricting the sub-region J to $E^\star$, the class of sub-rectangles with a corner at $\mathbf{0}$, i.e.

$$D_\infty^\star = \sup_{\mathcal{I} \subset E^\star} \left| \frac{1}{N} \#[\mathbf{x}^{(i)} \in \mathcal{I}] - \mathrm{Vol}(\mathcal{I}) \right|; \tag{S8}$$

$$(T_2^\star(N,d))^2 = \int\limits_{[0,1]^d} \left[\frac{1}{N} \#[\mathbf{x}^{(i)} \in [\mathbf{0},\mathbf{y}] - \mathrm{Vol}([\mathbf{0},\mathbf{y}]) \right]^2 d\mathbf{y}. \tag{S9}$$

In practice, it is typically not feasible to calculate the L_∞ measure (S6). Even in the case of OT, there are $[r(k+1)]^k$ sub-regions to consider. It is therefore common to use an L_2-based discrepancy, the main advantage

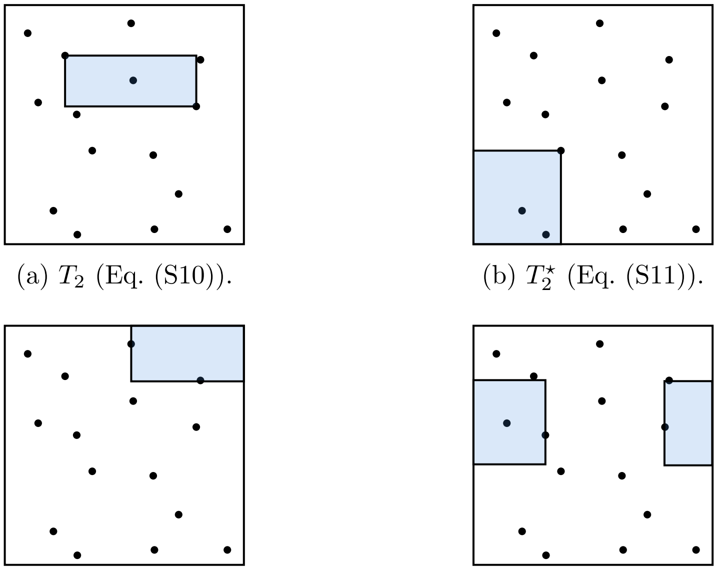

(a) T_2 (Eq. (S10)).

(b) $T_2^\star$ (Eq. (S11)).

(c) C_N (Eq. (S12)).

(d) W_N (Eq. (S13)).

Figure S1: Visual interpretation of different kinds of L_2 discrepancies. The blue-shaded area depicts the sub-region J for that discrepancy. Figure adapted from [2].

being that closed expressions are readily available (Eq. (S10)-(S13)).

$$T_2^2(N,d) = 12^{-d} + \frac{1}{N^2}\sum_{n=1}^{N}\sum_{m=1}^{N}\prod_{i=1}^{d}[1-\max(x_i^{(n)},x_i^{(m)})]\cdot\min(x_i^{(n)},x_i^{(m)}) - \frac{2^{1-d}}{N}\sum_{n=1}^{N}\prod_{i=1}^{d}x_i^{(n)}(1-x_i^{(n)}); \tag{S10}$$

$$(T_2^\star(N,d))^2 = 3^{-d} + \frac{1}{N^2}\sum_{n=1}^{N}\sum_{m=1}^{N}\prod_{i=1}^{d}[1-\max(x_i^{(n)},x_i^{(m)})] - \frac{2^{1-d}}{N}\sum_{n=1}^{N}\prod_{i=1}^{d}(1-(x_i^{(n)})^2); \tag{S11}$$

$$C_2^2(N,d) = \left(\frac{13}{12}\right)^d - \frac{2}{N}\sum_{n=1}^{N}\prod_{i=1}^{d}\left(1+\frac{1}{2}|x_i^{(n)}-\tfrac{1}{2}|-\frac{1}{2}|x_i^{(n)}-\tfrac{1}{2}|^2\right) + \frac{1}{N^2}\sum_{n=1}^{N}\sum_{m=1}^{N}\prod_{i=1}^{d}\left(1+\frac{1}{2}|x_i^{(n)}-\tfrac{1}{2}|+\frac{1}{2}|x_i^{(m)}-\tfrac{1}{2}|-\frac{1}{2}|x_i^{(n)}-x_i^{(m)}|\right); \tag{S12}$$

$$W_2^2(N,d) = -\left(\frac{4}{3}\right)^d + \frac{1}{N^2}\sum_{n=1}^{N}\sum_{m=1}^{N}\prod_{i=1}^{3d}\left(\frac{3}{2}-|x_i^{(n)}-x_i^{(m)}|(1-|x_i^{(n)}-x_i^{(m)}|)\right); \tag{S13}$$

Besides T_2 and $T_2^\star$, two other commonly used L_2 discrepancies are the centered (C_2) and wrap-around (W_2) discrepancy [3, 4, 5]. Figure S1 depicts the different kinds of sub-regions (i.e. restrictions of J) for each of the L_2-based discrepancies. W_2 discrepancy is less sensitive to boundary effects by wrapping the hypercube for each dimension [5]. For that reason, the wrap-around discrepancy is used in this work, since (E)OT and the 'pinned' methods naturally generate many parameter points on the boundary of the hypercube (i.e. $x_i = 0$ or $x_i = 1$). Indeed, from the closed expressions for T_N (Eq. (S10)) and $T_N^\star$ (Eq. (S11)) it is clear that they are not suitable for examining uniformity of such simulation point sets; by construction there is always at least one term in each product that will vanish if the number of levels $p = 4$ and the optimal step size $|\delta| = p/(2[p-1])$ are chosen.

Typically the expected discrepancy of a uniform random sample is used as a benchmark (see [2] for a closed expression for the W_2 benchmark); if the discrepancy of the QR sequence is significantly lower than the benchmark, the sequence is deemed good; if there is no significant decrease in discrepancy, or even an increase compared to the benchmark, the sequence is deemed poor. In all cases, the discrepancies of our point sets are much larger than the benchmark. In other words, our trajectories have a worse uniform coverage than a completely random sample. This is caused by the inherent clustering in the form of trajectories (OT) or stars (radial) and, in the case of OT or radial with integer/Boolean inputs, the fact that an input can only take one of p_i discrete values. One thus cannot use this benchmark to assess the quality of the set of trajectories.

S3 Sobol total sensitivity indices

Given that the factors are independent, the output variance $V(Y)$ for a model output Y with k scaled dimensionless input factors can be decomposed as [6]:

$$V(Y) = \sum_{i=1}^{k} V_i + \sum_{i=1}^{k} \sum_{j>i} V_{ij} + \ldots + V_{12\ldots k}, \tag{S14}$$

where the first two terms are given by

$$V_i = V_{X_i}(E_{\mathbf{X}_{\sim i}}[Y|X_i]); \tag{S15}$$

$$V_{ij} = V_{X_i X_j}(E_{\mathbf{X}_{\sim ij}}[Y|X_i, X_j]) - V_i - V_j, \tag{S16}$$

and the higher orders can be derived similarly [7]. Here $\mathbf{X}_{\sim i}$ denotes the mean is taken over all factors except X_i. V_i can be interpreted as the expected reduction in variance that would be obtained if X_i could be fixed. The associated sensitivity coefficients for the first two orders are [6]:

$$S_i = \frac{V_{X_i}(E_{\mathbf{X}_{\sim i}}[Y|X_i])}{V(Y)}; \tag{S17}$$

$$S_{ij} = \frac{V_{X_iX_j}(E_{\mathbf{X}\sim ij}[Y|X_i, X_j])}{V(Y)} - S_i - S_j; \tag{S18}$$

higher order indices are derived in a similar way. Note that these coefficients are normalized and sum to unity, i.e., $\sum_i S_i + \sum_i \sum_{j>i} S_{ij} + \ldots + S_{12\ldots k} = 1$. Alternatively, the *total effect index*, here also referred to as *Sobol total (sensitivity) index*, S_{Ti} measures the total effect, i.e. first order and interactions, of input X_i [6]. It is given by

$$S_{T_i} = \frac{E_{\mathbf{X}_{\sim i}}[V_{X_i}(Y|\mathbf{X}_{\sim i})]}{V(Y)} = 1 - \frac{V_{\mathbf{X}_{\sim i}}(E_{X_i}[Y|\mathbf{X}_{\sim i}])}{V(Y)}. \tag{S19}$$

One way of interpreting this quantity is by noting that $V_{\mathbf{X}_{\sim i}}(E_{X_i}[Y|\mathbf{X}_{\sim i}])$ is the first order effect of $\mathbf{X}_{\sim i}$, so $V(Y)$ minus this quantity must give the contribution of all terms in the variance decomposition which do include X_i (see [6, 7] for more detail). The total sensitivity index S_{T_i} is linked to the EE absolute mean effect $\mu_i^\star$ in the following way [7]. $\mu_i^\star$ is an approximation of the functional $\tilde{\mu}_i = \int_\Omega |\partial f/\partial x_i| d\mathbf{x}$, where f is the output of interest. In [7] it is shown that $S_{T_i} \leq C\tilde{\mu}_i/\pi^2 V$, where $|\partial f/\partial x_i| \leq C$ and V is the total variance of $f(\mathbf{x})$. Hence, small $\mu_i^\star$ imply small S_{T_i}. The reverse is not necessarily true[1], i.e. ranking factors based on S_{T_i} might give different results [7]. Nevertheless we expect that sampling strategies that are able to accurately estimate total sensitivity indices will also be able to accurately rank parameters (based on e.g. [9]).

Analytical values Sobol indices of test functions

The analytic S_{T_i} for the K-function (Eq. (41)) can be shown to equal

$$S_{T_i}(K) = \frac{\frac{1}{40}\left(\frac{1}{3}\right)^k + \frac{1}{8}\left(\frac{1}{3}\right)^i + \frac{1}{10}(-1)^{k+i}\left(\frac{1}{2}\right)^{k-i}\left(\frac{1}{3}\right)^i}{V(K)}, \tag{S20}$$

[1] Lower bounds on S_{T_i} in terms of $\tilde{\mu}_i$ do exist [8], but from these one cannot conclude that the ranking based on S_{T_i} is the same as the ranking found in EE.

with

$$V(K) = \frac{1}{10}\left(\frac{1}{3}\right)^k + \frac{1}{18} - \frac{1}{9}\left(\frac{1}{2}\right)^{2k} - \frac{2}{45}(-1)^k\left(\frac{1}{2}\right)^k. \tag{S21}$$

This expression is equivalent to the one given in [6], but in a more compact form.

Likewise, the analytical S_{T_i} for the G^*-function (Eq. (42) and Table 4) equal [6]:

$$S_{T_i}(G^*) = \frac{V_i \prod_{j\neq i}(1+V_j)}{\prod_{i=1}^{k}(1+V_i) - 1}, \tag{S22}$$

where

$$V_i = \frac{\alpha_i^2}{(1+2\alpha_i)(1+a_i)^2}. \tag{S23}$$

The analytical total sensitivity indices for the f_6-function (Eq. (49)) are [0.404365, 0.565778, 0.0148933, 0.012522, 0.002442, 0] [10].

Alternative numerical estimation of Sobol indices

A commonly used alternative to approximating the total variation $\hat{V}(Y)$ in Equation (51) is given by [11]:

$$\hat{V}(Y) = \frac{1}{r}\sum_{j=1}^{r}\left[Y(A_j) - Y_0\right]^2; \tag{S24}$$

$$Y_0 = \frac{1}{r}\sum_{j=1}^{r} Y(A_j). \tag{S25}$$

Here $Y(A_j)$ is the value of Y at the j-th base point and $Y(A_{B_j}^{(i)})$ is the value of Y at the perturbed value in the x_i-direction.

Differences in test setup with Saltelli et al.

Note that [6] does not divide by k in their definition of MAE (Eq. (54)), since in all their experiments $k = 10$. Furthermore, in [6] the input labels were randomly shuffled for the G^*-function (Eq. (42)), possibly to account for differences in uniform coverage of the QR sequence in higher dimensions. However, because we felt this potential effect is far outweighed by sampling the η_i's, we have not done this in our experiments. Finally note

that Saltelli et al. [6] plot ‘total cost’ on the horizontal axis, which is defined as $r(k+1)$ (regardless of the number of experiment replicates). To increase reproducibility, we show the number of trajectories per replicate in Figures 8-9.

Bibliography

[1] W. J. Morokoff and R. E. Caflisch. Quasi-random sequences and their discrepancies. *SIAM Journal on Scientific Computing*, 15(6):1251–1279, 1994.

[2] F. J. Hickernell. *What affects the accuracy of quasi-Monte Carlo quadrature?*, pages 16–55. Springer, 2000.

[3] K. Fang and C. Ma. Wrap-around L2-discrepancy of random sampling, Latin hypercube and uniform designs. *Journal of Complexity*, 17(4):608–624, 2001.

[4] K. Fang et al. Centered L2-discrepancy of random sampling and Latin hypercube design, and construction of uniform designs. *Math. Comput.*, 71(237):275–296, 2002.

[5] G. Damblin et al. Numerical studies of space filling designs: optimization of Latin Hypercube Samples and subprojection properties. *Journal of Simulation*, 7:276–289, 2013.

[6] A. Saltelli et al. Variance based sensitivity analysis of model output. design and estimator for the total sensitivity index. *Computer Physics Communications*, 181(2):259–270, 2010.

[7] I. M. Sobol' and S. Kucherenko. Derivative based global sensitivity measures and their link with global sensitivity indices. *Mathematics and Computers in Simulation*, 79(10):3009–3017, 2009.

[8] S. Kucherenko and S. Song. *Derivative-based global sensitivity measures and their link with Sobol' sensitivity indices*, pages 455–469. Springer, 2016.

[9] M. Lamboni et al. Derivative-based global sensitivity measures: general links with Sobol' indices and numerical tests. *Mathematics and Computers in Simulation*, 87:45–54, 2013.

[10] A. Puy et al. Is VARS more intuitive and efficient than Sobol' indices? *Environmental Modelling & Software*, 137:104960, 2021.

[11] A. Puy et al. The battle of total-order sensitivity estimators. *arXiv preprint arXiv:2009.01147*, 2020.
