# Supplementary material for: Elementary effects for models with dimensional inputs of arbitrary type and range: Scaling and trajectory generation
Source: PLoS One. 2023 Oct 25;18(10):e0293344. doi: 10.1371/journal.pone.0293344 (PMC10599590; doi:10.1371/journal.pone.0293344)
Supplement: S4 File — (PDF) [file pone.0293344.s004.pdf]

The following documents are part of the supplementary materials of “Elementary Effects for models with dimensional inputs of arbitrary type and range: scaling and trajectory generation”.

S1 Appendix: Elementary effects.
Definition that accounts for steps in the negative direction.

S2 Appendix: Discrepancy.
Information on discrepancy of a point set.

S2 Appendix: Sobol total sensitivity indices.
Information on Sobol total sensitivity indices, analytical values of Sobol total indices for the used test functions, and differences in test setup with Saltelli et al.

S4 Files: Code.
Code (in the XL language) used to obtain the numerical results.

S5 Files: Additional data and figures.
All data - including additional figures - in the form of Microsoft Excel spreadsheets.

S4 consists of two files:

1. elementaryEffects.rgg

   Contains the bulk of the code for executing an EE analysis. Please note this file is an .rgg file, which is an extension of Java (see Kniemeyer, 2008). We plan to apply the EE analysis to our functional-structural plant model, which runs on the GroIMP-platform (Hemmerling et al, 2008) which makes use of the RGG language. In practice, this file contains solely Java code, so potential users can freely copy/paste parts of the code for use in their own implementations.

   Further details on how to initiate and run this code can be found within the file.

2. EE_TestModel_main.rgg

   Main file for applying the different variants of the analysis to the test functions in the paper and establishes the connection with the GroIMP platform. This file contains GroIMP/RGG-specific syntax.

S5 These spreadsheets include a number of results that are not presented in the paper, such as the effect of a different shift in the QR-sequence.

References:

O. Kniemeyer. Design and Implementation of a Graph Grammar Based Language for Functional-Structural Plant Modelling. Doctoral thesis, 2008.

R. Hemmerling et al. The rule-based language XL and the modelling environment GroIMP illustrated with simulated tree competition. Functional Plant Biology,35:9–10, 2008.
