# Supplementary material for: Elementary effects for models with dimensional inputs of arbitrary type and range: Scaling and trajectory generation
Source: PLoS One. 2023 Oct 25;18(10):e0293344. doi: 10.1371/journal.pone.0293344 (PMC10599590; doi:10.1371/journal.pone.0293344)
Supplement: Attachment — Submitted filename: Cover letter - Copy.pdf [file pone.0293344.s005.pdf]

Dear Editor,

Many thanks for considering our manuscript ‘Elementary Effects for models with dimensional inputs of arbitrary type and range: scaling and trajectory generation’. We thank the reviewer for their consideration of this manuscript. We were delighted to see that the reviewer thought that “the novelty of the paper is adequate enough for possible publication”.

We have carefully considered the reviewer’s suggestions and addressed them as described in detail below. These suggestions have led us to revise the text in several places, including updating the literature review and further clarifying the most important contributions of our work. Changes to the text are highlighted in red. We hope that you find the revised manuscript suitable for publication.

Yours Sincerely,

Rik Rutjens

Reviewer #1:

1. **The novelty of the paper is adequate enough for possible publication at PLOS ONE.**

We appreciate the reviewer's appraisal of our work.

2. **Literature review should be strengthen using new published articles.**

The previously submitted version contains 60 relevant references. We feel that these references adequately describe the state of the field and the most important developments. We do however appreciate that there are a few recent papers that have not been included, in particular on alternative approaches to trajectory generation, that could be included in the paper. The following line has therefore been added to Subsection 2.4 of the manuscript: "*For a number of recent approaches (including cluster sampling), the reader is referred to \cite{EE_Feng2022, EE_Shi2023, EE_Shi_2021, EE_Wu2020, EE_Feng2020} and the references therein.*"

Should the reviewer still feel the literature review lacks key references, more specific direction from them would be helpful.

3. **The contribution and conclusion of the paper can better explore descriptively and quantitatively.**

To improve readability, we have removed or shortened non-essential lines throughout the manuscript, in particular in the results and conclusion sections (highlighted in red in the revised manuscript).
In addition, we have added quantitative statements (again highlighted in red) to the results paragraphs of Section 5.3 and 5.4, quantifying the difference in MAE between different trajectory generation methods and the differences in correlation coefficients between different combinations of trajectory generation methods and sensitivity indices. We believe the contributions and conclusions of the paper are now sufficiently supported by quantitative statements.

4. **Some remarks on main results would be necessary and helpful.**

We have attempted to improve highlighting of, and signposting to, the most important results (in line with the abstract), by making key statements in the conclusion boldface. To keep the manuscript a manageable length, we are wary of adding more text.
